# Researches on the Influence of Culinary Salt and Coffee on the Metamorphosis of Tissue

**Published:** 1863-05

**Authors:** 


RESEARCHES ON THE

### INFLUENCE OF CULINARY SALT AND COFFEE ON THE METAMORPHOSIS OF TISSUE.

Culnary salt, according to the researches of C. Voit, is a powerful stimulator of the metamorphosis of tissue; it increases, by means of its physical properties, the capillary circulation of fluids in the organism; it increases the oxidation of albumen, and through this the quantity of urea excreted. Culinary salt is also a true diuretic. In order to excrete the salt from the body, water is required; this water passes always through the kidneys (the only channel for the excretion of culinary salt in the dog,) and is, if the supply of water from without is limited, abstracted from the tissues.

Voit's experiments with *coffee* on a dog led to the inference that coffee does not, as is usually assumed, diminish the metamorphosis of nitrogenous tissue, and the excretion of urea, but, on the contrary, rather increases these processes. On the whole, the dog appeared to be more lively after the use of coffee. The author made also experiments with *caffein* on frogs, and found it to cause, at first, increased irritability of the nervous system, a tendency to reflex-movements and to tetanic convulsions; later, however, phenomena of paralysis. The pupil becomes dilated; the capillary vessels are filled with blood; the heart's contractions are at first increased, later reduced in frequency, they are arrested during the tetanic paroxysms. The author attributes the principal effects of coffee to its action on the nervous system, not to its influence on the tissue-change. The nervous system being rendered more susceptible, the same exciting cause produces a greater effect. Coffee thus refreshes, Voit thinks, the fatigued body, renders the lassitude less perceptible, and in this manner enables us to endure prolonged exertions. The experiments on the influence of *bodily exercise* (tread-wheel) on the tissue-change in the well-known dog lead to the unexpected result, that the excretion of urea was not at all, or only very slightly, increased by bodily labor. Voit infers, therefore, that muscular action does not cause increased decomposition of albuminous substances, while it is accompanied with a greater consumption of fat. As the decomposition of albumen is not the source of the production of force, connected with muscular contraction, Voit is inclined to look for it in the development of electricity.—*Brit. and For. Med. and Surg. Jour.*—*Drug Circ.*

